# Prediction of Suitable Habitats for *Bisetocreagris* (Pseudoscorpiones: Neobisiidae) in China and Its Application in Conserving Endemic Species Diversity

**DOI:** 10.1002/ece3.73436

**Published:** 2026-04-09

**Authors:** Shiqi Liu, Yingying Li, Fei Xie, Yunchun Li, Zhonghua Wei, Ding Yang, Aimin Shi

**Affiliations:** ^1^ The Key Laboratory of Southwest China Wildlife Resources Conservation of the Ministry of Education, College of Life Sciences China West Normal University Nanchong Sichuan Province China

**Keywords:** geographical distribution, MaxEnt, Pseudoscorpiones, zoogeography

## Abstract

Due to climate change damaging terrestrial ecosystems and threatening biodiversity, the conservation of cave‐endemic lineage has garnered growing attention, as they serve as unique model systems for studying climate sensitivity, distributional constraints, and extinction risk. Given the limited research on endemic species, this study aims to identify the limiting factors, distribution range, and future changes in suitable habitats for the endemic species *Bisetocreagris*, thereby providing a scientific basis for the conservation of cave ecosystems. We used 44 distribution points and a MaxEnt model to predict *Bisetocreagris* distribution, integrating seven environmental variables and three emission scenarios to assess climate‐driven habitat changes. The results indicate that factors such as Bio14, Bio2, and Bio7 collectively characterize the dependence of cave‐endemic lineages on stable microenvironments and the potential limits of their physiological tolerance, reflecting the niche specialization that has evolved in the genus *Bisetocreagris* during its long‐term adaptation to cave environments. Under current climate conditions, *Bisetocreagris* has a highly suitable growth area of 97.1877 × 10^4^ km^2^, which is primarily concentrated in southeastern China, with the widest distribution in Guizhou Province. Under various carbon emission scenarios, the potential suitable area consistently decreases. The high‐carbon‐emission SSP5–8.5 pathway has the most significant impact on high‐suitability area, resulting in a 97.8808% reduction by the 2090s compared to the present, leaving an area of only 2.0596 × 10^4^ km^2^. The narrow distribution ranges, specialized ecological niches, and limited dispersal capacity observed in *Bisetocreagris* are traits shared by many arachnids. The response patterns revealed in this study may therefore extend beyond the focal genus to reflect broader vulnerabilities within the class Arachnida. Consequently, this research not only provides a scientific basis for habitat conservation and priority area identification for *Bisetocreagris* but also offers a transferable framework for assessing climate change vulnerability across other arachnids.

## Graphical abstract

Using the MaxEnt model with 44 distribution points and seven key environmental variables, this study analyzed the potential habitats of the endemic species *Bisetocreagris* under three greenhouse gas emission scenarios, identified precipitation of the driest month (Bio14), mean temperature of the warmest quarter (Bio10), mean diurnal range (Bio2), and temperature annual range (Bio7) as critical influencing factors, and found that the species' current highly suitable habitat (97.1877 × 10^4^ km^2^, mainly in Guizhou Province) will shrink under all scenarios, thus highlighting climate change's adverse impacts and providing a scientific basis for the conservation of this endemic species.

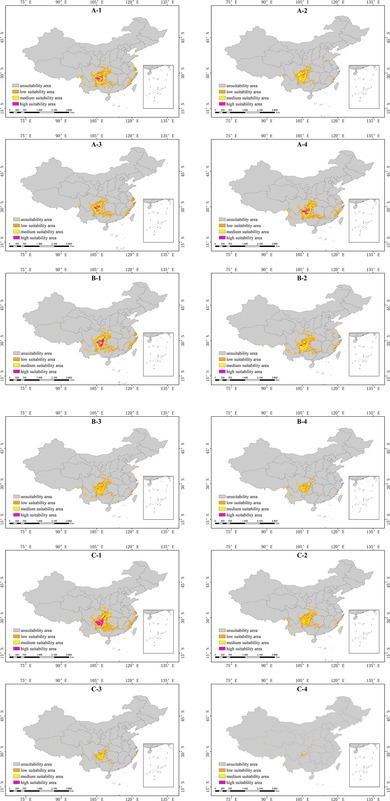

## 1 Introduction

The IPCC Sixth Assessment Report indicates that climate change has caused significant damage to terrestrial ecosystems and results in increasingly irreversible losses (Rinawati et al. 2013). Climate change significantly impacts biodiversity and ecosystems by altering species' physiological and ecological processes, geographical distribution patterns, and community interactions, thereby threatening the stability of ecosystem structure and function (Rinawati et al. 2013; Lenoir et al. 2008). Global warming induces changes in species' metabolic rates, triggering diverse survival and reproductive responses. Some species exhibit a sharp increase in population size, while others face the risk of local extinction (Fu et al. 2024; Mammola et al. 2018; Zhang et al. 2024). Invertebrates, as the most species‐rich group on Earth, play irreplaceable roles in maintaining ecological balance and biodiversity by undertaking key ecological functions such as material cycling and pollination. Additionally, they serve as research models to drive advancements across multiple disciplines, highlighting their unparalleled value in both ecological systems and scientific research (Verma et al. 2023; Filser et al. 2016). As one of the key functional groups among invertebrates, Arachnids are more significantly affected by climate change in terms of their survival and distribution, with distinct group‐specific responses (Mammola and Isaia 2017). Numerous studies have demonstrated that climate change profoundly impacts insect distribution ranges, affecting insect communities and their dispersal capabilities, which in turn influence the geographical distribution, population size, and growth of insects (Aidoo et al. 2022; Hu et al. 2025; Ma et al. 2021; Zhang et al. 2025). Therefore, it is essential to study the potential distribution areas of insects and the changing trends in their ecological ranges under climate change and formulate corresponding environmental management strategies.

Research on changes in species' potential suitable habitats requires scientific and effective models and methods, particularly those related to the Ecological Niche Models (ENMs) (Warren et al. 2021). ENMs—widely used in biodiversity conservation and climate change response research—are statistical approaches that integrate environmental variables like climate and terrain with species distribution data to predict species' potential suitable habitats or assess the impacts of environmental changes on their distributions (Warren et al. 2021; Elith and Leathwick 2009). The MaxEnt model is an algorithm based on the principle of maximum entropy and is often used to predict the potential distribution range of species (Phillips et al. 2006). The model analyzes the known distribution data of a species and a variety of environmental variables and constructs a model to infer the probability of occurrence of a species under different environmental conditions to map the potential habitat of a species (Phillips et al. 2006; Petitpierre et al. 2012). The MaxEnt model is one of the most widely used species distribution models because it is extremely flexible in terms of data requirements, demonstrates excellent model performance with small amounts of data, has high predictive accuracy and stability, and is relatively simple to use (Khan et al. 2022; Yang et al. 2022). The MaxEnt model is often used in conjunction with ArcGIS, playing a key role in predicting changes in species' habitats (Hosni et al. 2024; Rhodes and Sagan 2022).

Ecosystems encompass diverse biological communities on Earth and their interactions with the environment. As a unique type of ecosystem, cave systems are characterized by special environmental conditions, such as darkness, constant temperature, and high humidity, serving as habitats for many endemic species (Elith and Leathwick 2009; Howarth 1983). Among them, species of the Pseudoscorpiones order, as a key component of these systems, play a vital role in maintaining ecological balance. The genus *Bisetocreagris* belongs to the order Pseudoscorpiones, with 77 known species worldwide, 44 of which are cave species, all of which are endemic species. They not only inherit the climate‐sensitive trait of Arachnida but also superimpose the inherent vulnerability of cave‐dwelling lineage. This group provides a unique model system for studying species' climate sensitivity, distributional constraints, and extinction risk under climate change (Mammola et al. 2019).

Cave‐dwelling lineage, having long evolved in stable environments characterized by constant temperature and high humidity, is extremely sensitive to fluctuations in temperature and humidity. Their narrow physiological tolerance ranges make them valuable indicators for understanding species' sensitivity to climate change (Mammola et al. 2019; Vaccarelli et al. 2023; Sánchez‐Fernández et al. 2016). Due to the inherently fragmented nature and poor connectivity of cave habitats, coupled with the generally limited dispersal ability of cave‐dwelling lineage, their distribution patterns directly reflect the constraints of environmental factors on species ranges (Sánchez‐Fernández et al. 2018). Such restricted distribution increases the vulnerability of populations to extinction. As a result of small population sizes, limited gene flow, and weak buffering capacity against environmental change, cave‐dwelling lineage face significantly higher risks of local extinction compared to surface‐dwelling lineage (Mammola 2019; Grant et al. 2022). These traits of cave‐dwelling lineage are essentially adaptive outcomes shaped by long‐term stable environments. Local adaptation to cave microenvironments has led to the evolution of a strong dependence on stable temperature and humidity conditions, reflected in their narrow niche breadth. While this adaptive strategy confers survival advantages in stable cave habitats, it renders these species particularly vulnerable to external disturbances in the context of climate change (Mammola et al. 2019; Pallarés et al. 2020).

In addition, climate drying reduces the input of organic matter from the surface to caves, which directly threatens the food resources of *Bisetocreagris* (Bedoya‐Roqueme and Tizo‐Pedroso 2022; Harms et al. 2019). Furthermore, environmental changes caused by extreme precipitation and drought will further compress its habitat space. The cumulative effects of these factors make cave‐dwelling arachnids an ideal study system for examining the mechanisms by which climate change impacts biodiversity. This ultimately affects the distribution and population continuity of cave‐endemic lineages; therefore, conducting research on the potential suitable habitats of endemic species is particularly urgent (McClure et al. 2020). In the face of climate and environmental changes, it is particularly urgent to determine the potential suitable habitat of these species. Unique ecosystems represent a highly distinctive gene pool, with species being extremely fragile and susceptible to external influences (Mammola et al. 2019). For the research object from *Bisetocreagris*, which is small in size and has weak dispersal abilities, determining its suitable habitat not only enables the protection of endemic species, but it is also significantly important for the protection and management of cave ecosystems (Mammola et al. 2019; Rumpf et al. 2018; Thomas et al. 2004).

Due to global warming, many species are migrating to higher latitudes or elevations in search of optimal suitable areas (Lawlor et al. 2024). Species with strong dispersal ability have an advantage, as their distribution range can expand or shift, but species with weaker dispersal ability face the risk of extinction, leading to a reduction in biodiversity (Aidoo et al. 2022). Current research in this field mainly focuses on large, rare animals, with insufficient attention to smaller endemic species (Zhu et al. 2024; Roshani et al. 2024). Therefore, studying the potential suitable habitat of *Bisetocreagris* is urgent and needed. We hypothesize that climate change will result in a contraction of suitable habitat for *Bisetocreagris*, consequently reducing its overall distribution area and increasing the risk of local extinction, particularly for populations in marginal areas and shallow caves. This study is based on the current distribution points of the species and the environmental variables from global climate models and uses the MaxEnt model and ArcGIS 10.8. This study aims to address two core research questions: (1) What are the limiting factors and distribution ranges of *Bisetocreagris*? (2) How will the species' suitable habitat areas change in the 2030s, 2050s, 2070s, and 2090s?

## 2 Materials and Methods

### 2.1 Species Distribution Data

**FIGURE 1:**
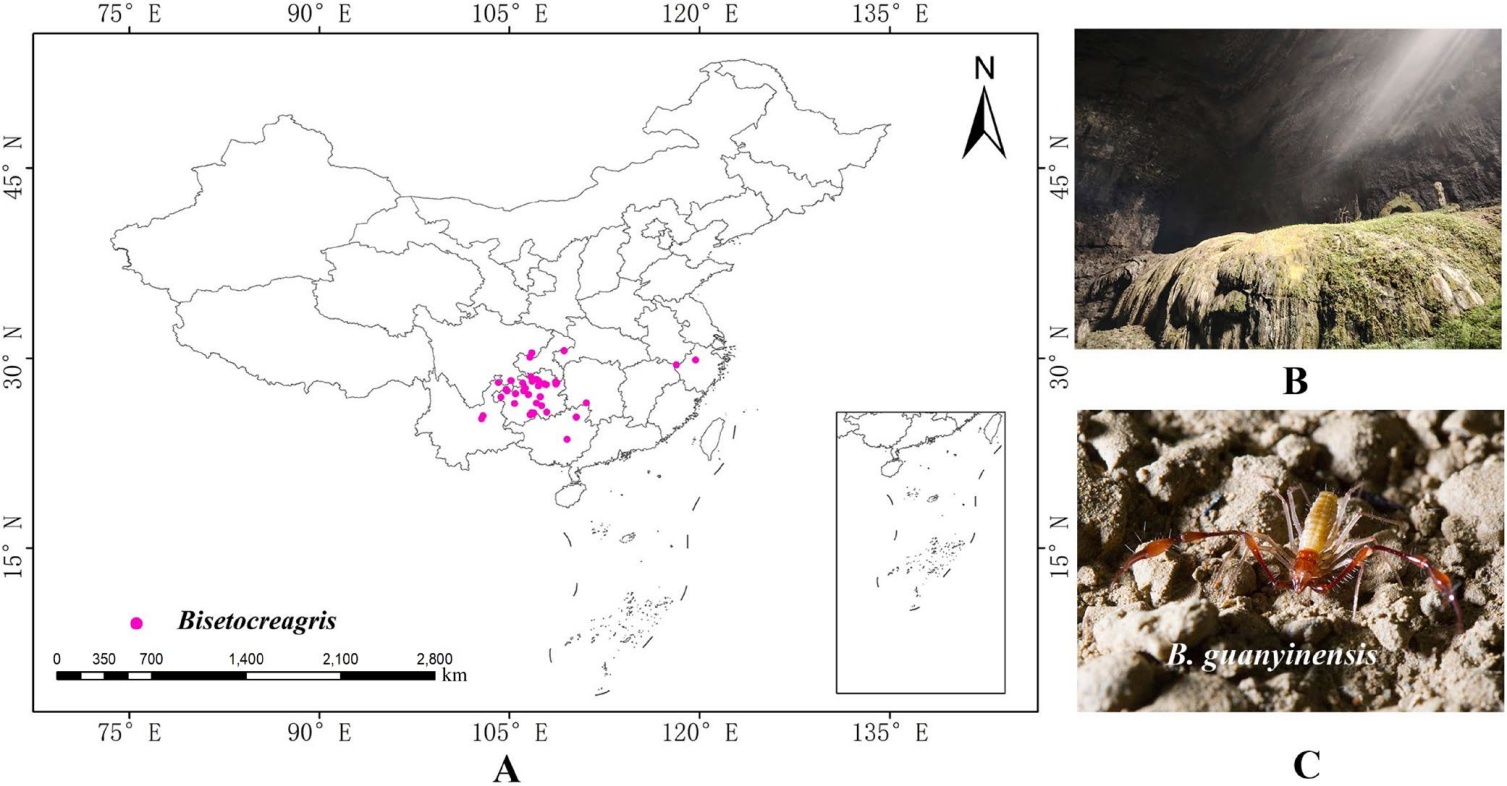
Distribution map, habitat, and field photograph of *Bisetocreagris guanyinensis* in China. (A) Distribution records; (B) Cave habitat at Tianyang Cave; (C) A *B. guanyinensis* individual at Guanyin Cave.

The data for *Bisetocreagris* in this study were sourced from the World Arachnida Catalog (https://wac.nmbe.ch/, accessed on 27 November 2025). During data collection, only distribution records with complete and accurate latitude and longitude coordinates were retained, while duplicate or erroneous sample points were excluded. To address the issue of spatial autocorrelation caused by overly close sample points, which could affect analytical accuracy, redundant data were filtered using ENM Tools software (Warren et al. 2021). A total of 44 valid distribution points of *Bisetocreagris* in caves were ultimately obtained for constructing the MaxEnt model, as shown in Figure 1A. Figure 1B shows the cave habitat at Tianyang Cave. The photograph of the *B. guanyinensis* individual at Guanyin Cave (Figure 1C) was kindly provided by Prof. Mingyi Tian.

### 2.2 Sources and Processing of Environmental Factors

The climate data for this study is sourced from the World Climate Data website (https://www.worldclim.org/, accessed on 23 November 2025), using data from the BCC‐CSM2‐MR model, as detailed in Table 1. It covers 19 environmental factors at five time points: the current period (1970–2020), the 2030s (2021–2040), the 2050s (2041–2060), the 2070s (2061–2080), and the 2090s (2081–2100), all with a spatial resolution of 2.5 min (Wei et al. 2023). For future periods, three representative concentration pathways are set as scenarios for greenhouse gas emissions: SSP1–2.6 (low emissions), SSP2–4.5 (medium emissions), and SSP5–8.5 (high emissions) (Riahi et al. 2017). The map data is sourced from the Resource and Environment Science and Data Platform (https://www.resdc.cn/, accessed on 23 November 2025).

**TABLE 1:** Contribution rates of environmental variables.

Climate factor	Environmental factor
Bio1	Annual Mean Temperature (°)
Bio2	Mean Diurnal Range (°)
Bio3	Isothermality (Bio2/Bio7) (×100)
Bio4	Temperature Seasonality (standard deviation ×100)
Bio5	Max Temperature of Warmest Month (°)
Bio6	Min Temperature of Coldest Month (°)
Bio7	Temperature Annual Range (BIO5–BIO6)
Bio8	Mean Temperature of Wettest Quarter (°)
Bio9	Mean Temperature of Driest Quarter (°)
Bio10	Mean Temperature of Warmest Quarter (°)
Bio11	Mean Temperature of Coldest Quarter (°)
Bio12	Annual Precipitation (mm)
Bio13	Precipitation of Wettest Month (mm)
Bio14	Precipitation of Driest Month (mm)
Bio15	Precipitation Seasonality (mm)
Bio16	Precipitation of Wettest Quarter (mm)
Bio17	Precipitation of Driest Quarter (mm)
Bio18	Precipitation of Warmest Quarter (mm)
Bio19	Precipitation of Coldest Quarter (mm)

During the data preprocessing stage, the 19 environmental factor datasets were converted to ASCII format using ArcGIS v10.8 geographic information system software. To mitigate the interference of environmental factor multicollinearity on model performance and avoid overfitting, a stepwise selection strategy was employed. First, Pearson correlation coefficients between each pair of environmental factors were calculated in ENM Tools, with variables having an absolute value greater than 0.8 considered strongly correlated (Yunsheng 2007). Then, the Jackknife method in MaxEnt v3.4.1 software was used to evaluate the contribution of the 19 environmental factors to the model, removing those with a contribution rate of less than 1% and retaining those with higher contribution rates within strongly correlated variable groups (Chao et al. 2020). Finally, seven environmental factors (Bio14, Bio10, Bio2, Bio7, Bio19, Bio12, and Bio15) were selected for constructing the ecological niche model of *Bisetocreagris*.

### 2.3 MaxEnt Model Construction

Model calibration was performed on the 7 selected climate factors using the kuenm package in R v.4.3.2 software, with the step size set to 0.5 to ensure model accuracy (Cobos et al. 2019). The model performance and complexity were evaluated using the omission rate and the corrected Akaike Information Criterion (AICc), respectively (Cobos et al. 2019). In this study, MaxEnt software was utilized for model construction. The distribution records of *Bisetocreagris* were organized in CSV format and imported into the software along with preprocessed environmental variables. The output type was set as Logistic, with 75% of the species distribution data allocated for model training and 25% reserved as the test set. The maximum number of iterations was 5000, the regularization multiplier was set to 0.5, and the model used a combined feature form of hinge features and product features, while other parameters remained at their default values (Yan et al. 2020). The Jackknife method was employed to assess the contribution of each factor to the model, followed by performance evaluation using the receiver operating characteristic (ROC) curve. The horizontal coordinate of the ROC curve represents the rate of actual absence but predicted presence, and the vertical coordinate represents the rate of actual presence and predicted absence. The area enclosed by the ROC curve and these two coordinates is called the AUC. The receiver operating characteristic (ROC) curve and the area under the curve (AUC) are used to evaluate the prediction accuracy. The AUC value ranges from 0 to 1. The higher the AUC value, the higher the accuracy of the prediction results. Specifically, 0.5 to 0.6 indicates failure of the results, 0.6 to 0.7 indicates poor results, 0.7 to 0.8 indicates fair results, 0.8 to 0.9 indicates good results, and 0.9 to 1.0 indicates very good results (Sun et al. 2023).

### 2.4 Suitable Habitat Zonation

ArcGIS v10.8 software was used to visualize the output of the MaxEnt model and analyze habitat suitability. Jenks Optimization is employed to categorize the habitat into one of four categories: unsuitability area (0–0.07), low‐suitability area (0.07–0.24), medium‐suitability area (0.24–0.49), and high‐suitability area (0.49–1) (Zhang et al. 2024).

## 3 Results

### 3.1 Model Accuracy Evaluation

**FIGURE 2:**
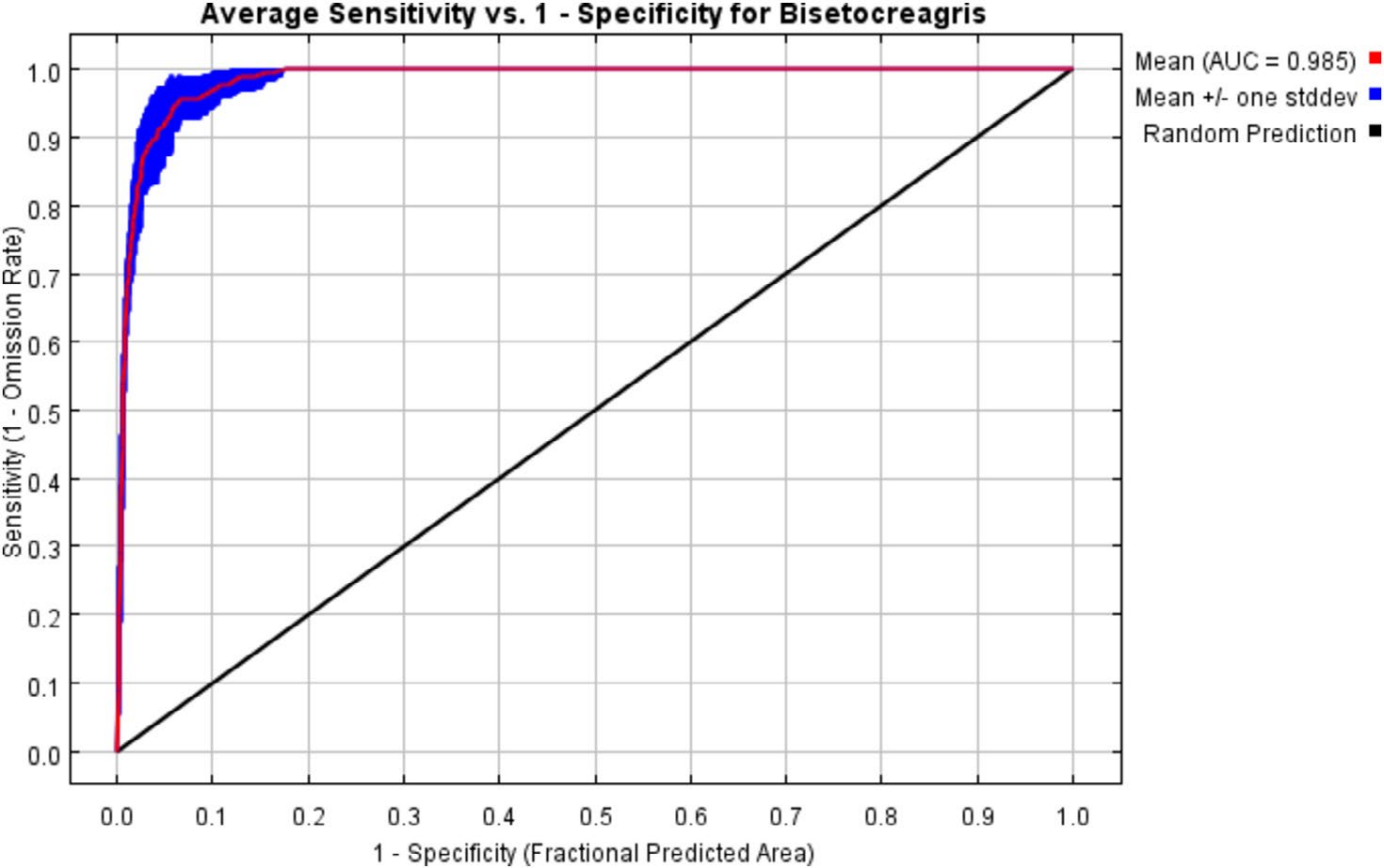
Receiver operating characteristic (ROC) curve of *Bisetocreagris*.

Following 10 iterations of cross‐validation, the MaxEnt model's predictive results, as indicated by the ROC curve, yielded an average AUC of 0.985 for the training set and the standard deviation is 0.005 (Figure 2), demonstrating a very high level of reliability in predicting the potential suitability areas of *Bisetocreagris*.

### 3.2 Key Climate Factors Influencing *Bisetocreagris* Distribution

This study calculated the contribution of each environmental variable using MaxEnt software. The results showed that precipitation of driest month (Bio14) made the largest contribution to model construction, accounting for 55.7%, highlighting the species' high sensitivity to precipitation. This was followed by mean temperature of warmest quarter (Bio10), mean diurnal range (Bio2), and temperature annual range (Bio7), which contributed 16.1%, 9.8%, and 9.4%. Precipitation of the coldest quarter (Bio19), annual precipitation (Bio12), and precipitation seasonality (Bio15) followed, while these three variables had minimal significant effects on the species, as detailed in Table 2.

**TABLE 2:** Percent contribution and permutation importance of variables in the MaxEnt model.

Climate factor	Environmental factor	Percent contribution (%)	Permutation importance (%)
Bio14	Precipitation of Driest Month (mm)	55.7	35.6
Bio10	Mean Temperature of Warmest Quarter (°)	16.1	4.1
Bio2	Mean Diurnal Range (°)	9.8	21.1
Bio7	Temperature Annual Range (BIO5–BIO6)	9.4	20.7
Bio19	Precipitation of Coldest Quarter (mm)	5.2	9.3
Bio12	Annual Precipitation (mm)	2.5	5.3
Bio15	Precipitation Seasonality (mm)	1.3	3.8

**FIGURE 3:**
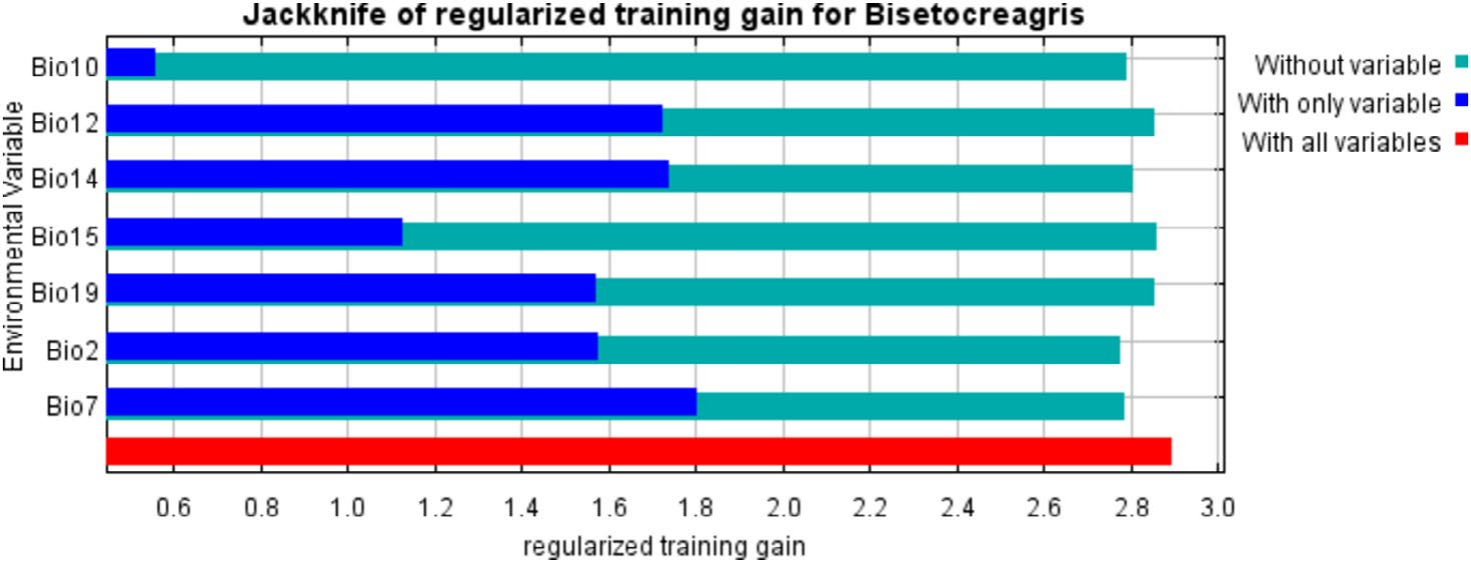
Assessing the importance of environmental variables affecting the distribution of *Bisetocreagris* using the Jackknife test.

The results of the Jackknife test (Figure 3) indicate that the seven environmental variables all influence the distribution of suitable habitats for *Bisetocreagris* to varying degrees. Among all climate factors, precipitation and temperature play critical roles in predicting the species' potential distribution. In single‐variable tests, temperature annual range (Bio7) showed the highest gain value, confirming its dominant impact on *Bisetocreagris* distribution, followed by precipitation of driest month (Bio14), annual precipitation (Bio12), mean diurnal range (Bio2), and precipitation of coldest quarter (Bio19). Moreover, when excluding Bio2 from the model, the gain value dropped to the lowest level, demonstrating that Bio2 contains unique information not captured by other factors and is thus a key driver in the model. This was followed by Bio7, Bio10, and Bio14, which also exhibited significant independent contributions.

**FIGURE 4:**
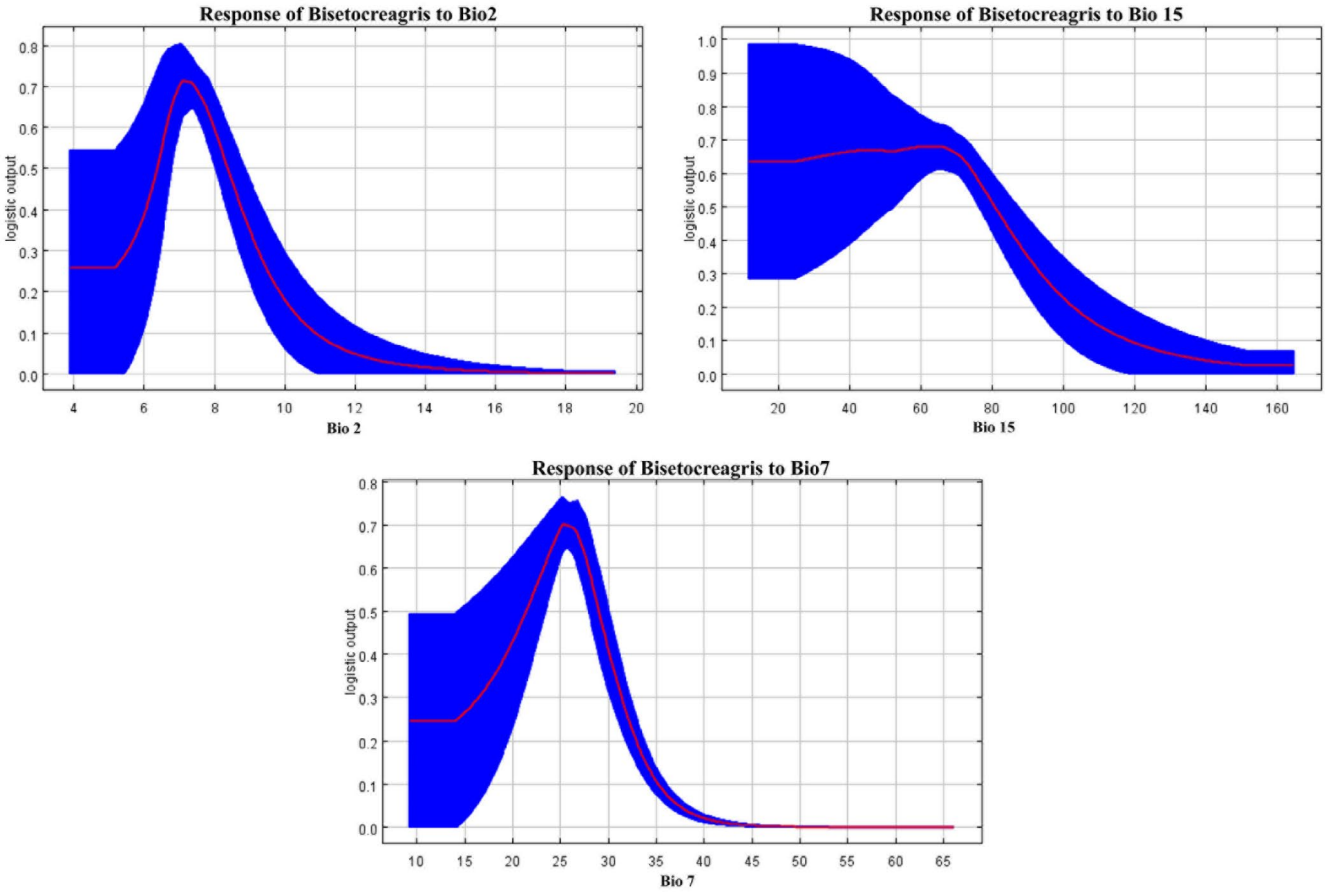
Response curves for dominant environmental factors.

The response curves of the dominant environmental variables for *Bisetocreagris* were obtained using the MaxEnt model (Figure 4). It can be observed from the figure that for the suitable living environment of *Bisetocreagris*, the suitable range of precipitation seasonality (Bio7) is approximately between 21°C and 29°C, with the most suitable precipitation value being around 25°C; the suitable range of precipitation seasonality (Bio15) is roughly 11% to 81%, with the most suitable value being close to 63%; and the suitable range of mean diurnal range (Bio2) is approximately between 6°C and 8°C, with the most suitable temperature value being around 7°C.

### 3.3 Distribution of Suitable Habitats for *Bisetocreagris* Under the Current Climate

**FIGURE 5:**
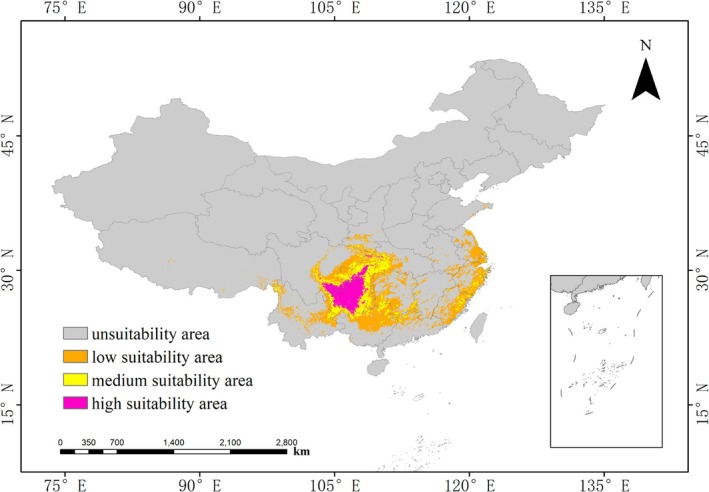
Current suitable distribution of *Bisetocreagris* in the world.

The current suitability area distribution map of *Bisetocreagris* was generated using the MaxEnt model (Figure 5). Under current environmental conditions, the total area of suitable habitat for *Bisetocreagris* is 97.1877 × 10^4^ km^2^, accounting for approximately 10.2334% of China's total. Within this area, the high‐suitability area is relatively small, spanning 13.6963 × 10^4^ km^2^ and only accounting for 14.0926% of the total suitability area, and is primarily distributed in Sichuan Province, Chongqing Municipality, and Guizhou Province. The medium‐suitability area covers 21.4516 × 10^4^ km^2^, 22.0724% of the total habitat of this species, extending across Guizhou Province, Chongqing Municipality, Hubei Province, and Sichuan Province. The low‐suitability area is the largest, comprising 62.0398 × 10^4^ km^2^, accounting for 63.8350% of the total suitability area, and is distributed in Guangxi Zhuang Autonomous Region, Hunan Province, Zhejiang Province, Fujian Province, and Jiangsu Province. Overall, *Bisetocreagris* has its most concentrated suitable habitats in southwestern China, with the largest low‐suitability area spanning multiple provinces.

### 3.4 Potential Suitable Habitat Distribution of *Bisetocreagris* Under the Future Climate

Under the context of future climate change, this study selected four time periods and constructed environmental scenarios based on three different Shared Socioeconomic Pathways (SSPs). The MaxEnt model was used to generate the distribution map of potential suitable habitats for *Bisetocreagris* (Figure 5). Additionally, the areas under different scenarios in each period were obtained, with detailed data presented in Table 3. The results show that the area of potential suitable habitats for *Bisetocreagris* will continue to decline compared with the current area. As shown in Table 3, under the SSP1–2.6 climate scenario, the suitability area will decrease across all periods, with the largest reduction occurring in the 2050s and decreasing by 65.3053% to 33.7190 × 10^4^ km^2^. The high‐suitability area will also decrease under this scenario, with the most significant reduction occurring in the 2050s and amounting to 0.8681 × 10^4^ km^2^, a 93.6618% decrease compared to the current area. The low‐suitability area will also decline, with the most pronounced reduction occurring in the 2050s and the area of suitable habitats decreasing to 22.6991 × 10^4^ km^2^, a reduction of 63.4120%. The medium‐suitability area will also decrease, with the most significant reduction occurring in the 2070s, accounting for only 56.9020% of the current area. Under the medium‐emission (SSP2–4.5) scenario, the potential suitability area for *Bisetocreagris* will decrease, and by the 2090s, compared to the current period, the areas of all three suitability classes will shrink to varying degrees. Under the SSP5–8.5 scenario, the change in the suitable habitat area of *Bisetocreagris* will be the most significant, with the areas of all three suitability classes decreasing gradually. By the 2090s, the total suitable habitat area will be only 2.0596 × 10^4^ km^2^, representing a 97.8808% reduction compared with the present. In this period, the low‐suitability area will decrease by 96.8256%, the medium‐suitability area by 99.5851%, and the high‐suitability area by as much as 99.9912%, its remaining area merely 0.0012 × 10^4^ km. Overall, the change in the potential suitability area of *Bisetocreagris* is closely related to emission scenarios and time. Under the high‐emission scenario, the total suitability area will show the most significant change in the 2090s, with a reduction of 97.8808%.

**TABLE 3:** Current and future suitable areas of *Bisetocreagris*.

Period	Scenarios	Low suitability (×10^4^ km^2^)	Medium suitability (×10^4^ km^2^)	High suitability (×10^4^ km^2^)
Current	—	62.0398	21.4516	13.6963
2030s	SSP1–2.6	37.2685	12.6492	4.4356
	SSP2–4.5	36.0598	14.3898	6.7261
	SSP5–8.5	40.6943	14.1648	8.7482
2050s	SSP1–2.6	22.6991	10.1518	0.8681
	SSP2–4.5	34.3556	12.2532	1.8377
	SSP5–8.5	30.0878	8.7773	0.4969
2070s	SSP1–2.6	31.3815	9.2452	3.1425
	SSP2–4.5	27.1626	9.1441	0.9043
	SSP5–8.5	14.5819	4.1270	0.0342
2090s	SSP1–2.6	36.7865	12.1869	4.6024
	SSP2–4.5	16.9786	4.8518	0.2388
	SSP5–8.5	1.9694	0.0890	0.0012

**FIGURE 6:**
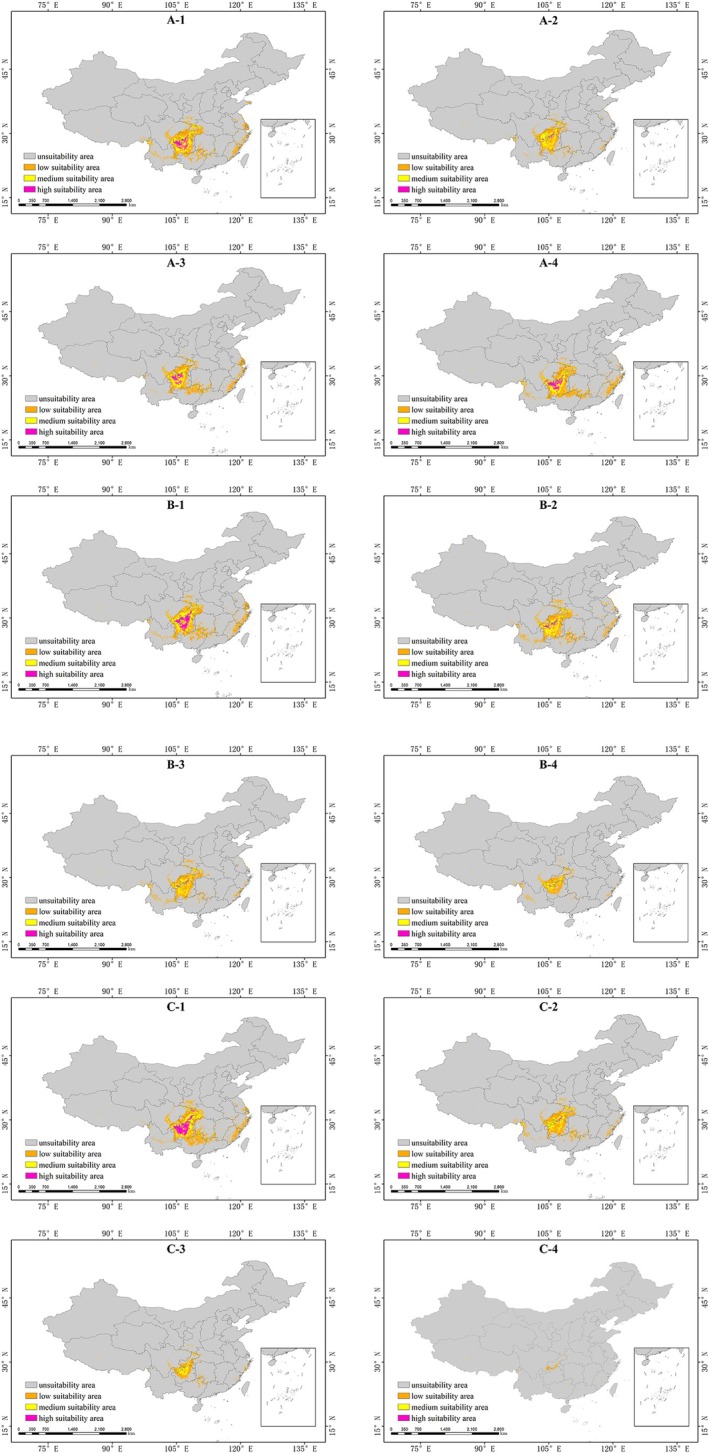
Potential distribution of *Bisetocreagris* in future periods (2030s, 2050s, 2070s, and 2090s) under the SSP1‐2.6, SSP2‐4.5, and SSP5‐8.5 climate change scenarios. In the figure, letters A, B, and C correspond to the SSP1‐2.6, SSP2‐4.5, and SSP5‐8.5 pathways, respectively, while numbers 1 to 4 represent the 2030s through the 2090s in sequence.

Analysis of the potential suitable habitat distribution map (Figure 6) for *Bisetocreagris* reveals a general trend in shrinking suitability area, with particularly significant changes observed in Guizhou Province. Under the high‐carbon‐emission scenario (SSP5–8.5), the distribution pattern of potential suitable habitats undergoes drastic changes: the area of high‐suitability habitats in Guizhou Province shrinks sharply, and the range of the original dominant suitable habitats also decreases significantly. In contrast, under the low‐emission scenario (SSP1–2.6), the potential suitable habitat area experiences the least reduction, indicating that low‐carbon development pathways help maintain the stability of suitable habitats. From a long‐term evolutionary perspective, the suitable habitats of *Bisetocreagris* are undergoing remarkable changes, with a particularly obvious downward trend in the area of suitable habitats in inland regions.

## 4 Discussion

The optimal model selected in this study adopts the qp feature combination with RM of 0.5, and its performance is verified comprehensively through multiple indicators: the model's 5% omission rate is at a low level, combined with an extremely high AUC value, indicating that it can accurately capture the known distribution of *Bisetocreagris* (Liu et al. 2013). For *Bisetocreagris*, this effectively avoids the omission of key habitats, highlighting the significance of the prediction. Meanwhile, the model's AICc performed best among all parameter combinations, providing statistical support for the accurate characterization of the species' distribution pattern (Ye et al. 2021). The hp. feature enhances the ability to characterize responses to complex habitats. Although a low value of RM weakens the penalty for complex structures, combined with the low omission rate and stable AICc results, it shows that the model retains ecological details while achieving reasonable control over complexity (Liu et al. 2022). This provides a reliable basis for analyzing the ecological association between *Bisetocreagris* and caves.

One limitation of this study lies in the singularity of its data source. To ensure maximum accuracy in species identification and locality records, this study adopted data from the World Arachnida Catalog (WAC) and did not include records from other databases such as the Global Biodiversity Information Facility (GBIF). Although the GBIF contains extensive citizen science and museum digitization data that could theoretically increase sample size, preliminary screening of *Bisetocreagris* records revealed issues with inconsistent geographic coordinate precision in some entries. Therefore, after weighing data breadth against data accuracy, we considered the current analyses based on the WAC to be more robust at the scale of this study. This limitation inevitably constrains the model's transferability and predictive stability to some extent. Other studies indicate that prediction accuracy improves non‐linearly with increasing sample size before stabilizing (Ye et al. 2021). Thus, future research should integrate data from multiple sources with stricter quality control procedures to enhance the model's generalization ability and predictive reliability.

This study used meteorological station data to model the distribution of cave‐dwelling species and revealed the relationship between regional climate and species distribution. Since meteorological data reflect surface macroclimate, which differs greatly from cave microclimate, prediction accuracy may be affected (Klinges et al. 2025). Despite these limitations, this study provides a macro‐level perspective for understanding regional distribution patterns of cave‐dwelling lineages and reveals the constraining role of broad‐scale climatic factors. Future research could integrate cave microclimate monitoring, species' ecological traits, and vertical climate gradient models to provide methodological references for studying climate responses of cave ecosystems in data‐scarce environments.

Under current climatic conditions, the total suitable habitat area for *Bisetocreagris* is 97.1877 × 10^4^ km^2^, with its most suitable habitats predominantly concentrated in Guizhou Province. The distribution of these high‐suitability areas aligns with the preferences of *Bisetocreagris* within cave environments. Previous studies suggest that high‐suitability zones may harbor rich genetic diversity and serve as core regions for species germplasm resource distribution. Therefore, it is recommended to prioritize surveying, resource collection, and conservation efforts in these areas. Under future climate change scenarios, the high‐carbon emission pathway (SSP5–8.5) has the most pronounced impact on the high‐suitability areas of *Bisetocreagris*, causing more significant changes in suitable habitat area compared to the other two pathways. This is because high carbon emissions exacerbate global warming, leading not only to rising temperatures but also to altered precipitation patterns. *Bisetocreagris* is highly sensitive to temperature and precipitation, which drastically reduces its suitable habitats. Although a small number of new suitable habitats may emerge, the overall quality of habitats will decline, and *Bisetocreagris* has weak migration ability (Mammola 2019). If current trends persist, the most suitable habitat areas for *Bisetocreagris* may decrease or even disappear.

In the process of modeling the environmental factors influencing the distribution of *Bisetocreagris*, precipitation contributes the most (64.7%) among the seven considered factors, with the remaining contributions coming from temperature‐related variables, indicating that precipitation is the primary driver of the species' distribution. The results of this study confirm the significant impact of precipitation on the distribution and growth of *Bisetocreagris*. As an important component of arachnids, this response pattern observed in *Bisetocreagris* is not an isolated phenomenon. Among arachnids, precipitation has also been demonstrated to be a key environmental driver. Previous studies have established strong links between precipitation and the geographic distribution as well as individual development of arachnids. This consistency indicates that climatic conditions associated with precipitation are critical not only for specific groups such as *Bisetorcreagris* but also serve as a general driving force shaping the biogeographic patterns of arachnids as a whole (Kusch 2015; Dang and Chen 2011; Li et al. 2020). Precipitation of Driest Month (Bio14) contributed most significantly, indicating that precipitation during the driest period is an important factor limiting the distribution of this species (Meehan et al. 2020). This result is highly consistent with previous observations in Andean soil fauna communities. During the driest period, soil fauna richness and abundance reach their lowest annual levels, and species in the surface litter layer migrate to deeper soil layers to avoid dry conditions. For *Bisetocreagris*, when the buffering capacity of caves or deep soil layers is insufficient to offset extreme drought stress and their dispersal ability is weak, local populations face a higher risk of extinction (Meehan et al. 2020; Castillo‐Figueroa and Castillo‐Avila 2025).

Importantly, the influence of seasonal precipitation is not independent; it also interacts closely with temperature‐related factors. Mean Diurnal Range (Bio2) is the influential environmental factor for this species. This factor forces *Bisetocreagris* to frequently adjust its habitat selection and activity patterns to avoid extreme temperatures, thereby increasing metabolic expenditure (Raschmanová et al. 2018). This phenomenon is consistent with survival patterns observed in other arachnids. In environments characterized by large temperature fluctuations, soil layers serve as critical refugia due to their relatively stable thermal conditions. During cold periods, species can move downward into warmer soil layers to avoid low temperatures. Under overheating conditions, a similar mechanism allows them to buffer the direct physiological stress caused by external temperature fluctuations (Castillo‐Figueroa et al. 2024). This microhabitat selection behavior reflects the direct response of arachnids to temperature variability (Adis et al. 2001). Temperature Annual Range (Bio7) affects the activity rhythms and population abundance of prey taxa. Unstable temperature conditions can significantly reduce prey abundance, while near‐freezing low temperatures impose direct physiological stress on prey populations. This factor also regulates the overall population size and vertical distribution of prey (Castillo‐Avila et al. 2025). Although extreme high temperatures can severely reduce prey abundance, and some taxa may mitigate unfavorable conditions through vertical migration, the overall stability of food supply remains disrupted. These effects increase the energy expenditure and adaptation costs for the species. These physiological and behavioral responses observed in *Bisetorcreagris* likely reflect a broader adaptive syndrome among arachnids, particularly those inhabiting soil ecosystems. Many soil‐dwelling arachnids have limited dispersal abilities and a strong dependence on microhabitat stability, making them vulnerable to similar selective pressures from temperature and precipitation variability (Castillo‐Avila et al. 2025; Briones et al. 1997). Consequently, the combined effects of climate variability, mediated through both direct physiological constraints and indirect trophic interactions, may represent a general driver of population dynamics and community structure in soil fauna (Zhang et al. 2014).

These research findings indicate that *Bisetocreagris*, as an endemic cave‐dwelling lineage, is highly dependent on the cave microenvironment. Habitat conditions and ecological requirements are species‐specific, making members of *Bisetocreagris* disproportionately vulnerable to climate change and human disturbance in China. This pattern is highly consistent with findings from studies on whip‐scorpions, which are also particularly sensitive to environmental change as a result of habitat specialization and narrow distribution ranges (Castillo‐Figueroa et al. 2024). Placing the results of this study within the broader context of arachnid ecology helps deepen our understanding of how cave‐specialized lineages respond to global change. Facing the dual pressures of human activity disturbances and low attention to the ecosystem, it is at risk of habitat fragmentation and population decline (Monro et al. 2018). To protect this endemic species, it is necessary to prioritize the protection of caves at known distribution sites, prevent cave desiccation or pollution from mining and groundwater extraction, restrict human access to avoid man‐made damage to the cave microclimates, and classify cave location information as sensitive data to prevent looting of cave resources or illegal collection of cave organisms after disclosure (Marsh et al. 2023). The legislative process for cave reserves must also be expedited, surrounding human activities strictly regulated, habitat restoration and ecological monitoring supported through special funds, the International Union for Conservation of Nature (IUCN) scientific assessments used to dynamically refine the protection list, and public education strengthened to raise social awareness of cave species conservation (Mammola et al. 2019; Duan et al. 2021). Furthermore, continuous monitoring of temperature and humidity changes in the cave microclimates amid global warming is essential. The assessment of their potential impacts on populations and the development of targeted measures when necessary are also essential (Hughes et al. 2023). Future studies should explore population dynamics monitoring technologies, quantify the impact thresholds of human activities, and assess the long‐term effectiveness of strategies using ecological models (McClure et al. 2020). In addition, strengthening international cooperation and interdisciplinary research will advance the process of biodiversity protection in caves.

## 5 Conclusions

This study employed a MaxEnt model to analyze the primary environmental factors influencing the current distribution of *Bisetocreagris*, predict its potential geographical distribution under climate change, and identify its suitability area in both present and future climate scenarios. The study results indicate that the primary environmental factors influencing the total distribution of *Bisetocreagris* in China are precipitation of driest month (Bio14), mean temperature of warmest quarter (Bio10), mean diurnal range (Bio2), and temperature annual range (Bio7). Currently, the total area of suitable habitat for *Bisetocreagris* is 97.1877 × 10^4^ km^2^, which is primarily distributed in Guizhou Province. Under the climate change scenario, the high emissions pathway significantly impacts the area of its suitable habitat, reducing it by 97.8808% compared to the current area. In the future, the area of suitable habitat for *Bisetocreagris* will decrease compared to the current situation, indicating that climate change will negatively impact this species in the coming years. As representative specialized predators in cave ecosystems, the vulnerability of *Bisetocreagris* may reflect a broader ecological pattern. Other arachnid lineages that share similar ecological niches with *Bisetocreagris* also face the risk of habitat loss due to climate change. Therefore, this study is not only significant for the conservation and ecological research of the endemic genus *Bisetocreagris* but also provides important references for identifying conservation priorities for cave‐dwelling arachnids. This contributes to an essential foundation for achieving biodiversity conservation and sustainable development.

## Author Contributions

**Shiqi Liu:** data curation (equal), software (equal), validation (equal), visualization (equal), writing – original draft (equal). **Yingying Li:** validation (equal), visualization (equal). **Fei Xie:** data curation (equal), software (equal). **Yunchun Li:** formal analysis (equal), investigation (equal), methodology (equal), project administration (equal), resources (equal), supervision (equal), writing – original draft (equal), writing – review and editing (equal). **Zhonghua Wei:** investigation (equal), methodology (equal), writing – review and editing (equal). **Ding Yang:** conceptualization (equal), funding acquisition (equal), resources (equal), supervision (equal). **Aimin Shi:** conceptualization (equal), funding acquisition (equal).

## Funding

The authors have nothing to report.

## Conflicts of Interest

The authors declare no conflicts of interest.

## Data Availability

The data supporting this study are openly available in the Dryad Digital Repository at https://doi.org/10.5061/dryad.3r2280gxb upon acceptance.
